# Spontaneous rupture of a giant gastrointestinal stromal tumor of the jejunum: a case report and literature review

**DOI:** 10.1186/1477-7819-12-153

**Published:** 2014-05-21

**Authors:** Shun-ichi Misawa, Misuzu Takeda, Hiroto Sakamoto, Yasushi Kirii, Hiroyoshi Ota, Hiroyuki Takagi

**Affiliations:** 1 Department of Surgery, Matsumoto City Hospital, 4417-180 Hata, Matsumoto, Nagano 390-1401, Japan; 2 Department of Biomedical Laboratory Sciences, Shinshu University, School of Medicine, School of health Science, 3-1-1 Asahi, Matsumoto, Nagano 390-8621, Japan

## Abstract

A few cases of a gastrointestinal stromal tumor (GIST) of the small intestine presenting as rupture have been reported in the medical literature. We report an unusual case of a large GIST of the jejunum that presented as a spontaneous rupture. A 70-year-old man was referred to our hospital because of fever and abdominal pain. An abdominal enhanced computed tomography (CT) scan detected a 10-cm tumor with heterogeneous staining, suggesting necrosis or abscess inside the tumor. The patient was treated with antibiotics but inflammation persisted and an operation was performed. Intraoperative findings showed an outgrowing 10-cm mass in the jejunum near Treitz's ligament. The tumor had ruptured with peritoneal metastasis. The solid parenchyma contained a focal area of necrosis within and the small ulcer located in the wall of the jejunum presented a communication with the large tumor cavity. H&E staining showed spindle-shaped cell proliferation, and immunohistochemical staining showed results positive for c-kit and CD34. The patient received a diagnosis of a GIST (high-risk group) of the jejunum and was treated with imatinib mesylate. The patient has remained in good health without recurrence or metastasis one year after the surgical procedure.

## Background

Gastrointestinal stromal tumors (GISTs) are the most common mesenchymal tumor of the gastrointestinal tract [1]. Most GISTs are > 5 cm in diameter at the time of diagnosis, with a diameter of 10 cm being associated with a higher risk of local or distant metastasis. In addition to tumor size, mitotic rate and tumor location, tumor rupture is thought to be a prognostic factor for the outcome of patients with a GIST. Gastrointestinal bleeding is the most common presentation (50%) of GISTs and is usually associated with ulceration of the tumor into the lumen [2,3]. We report an unusual case of a large GIST of the jejunum that presented as spontaneous rupture.

## Case presentation

**Figure 1:**
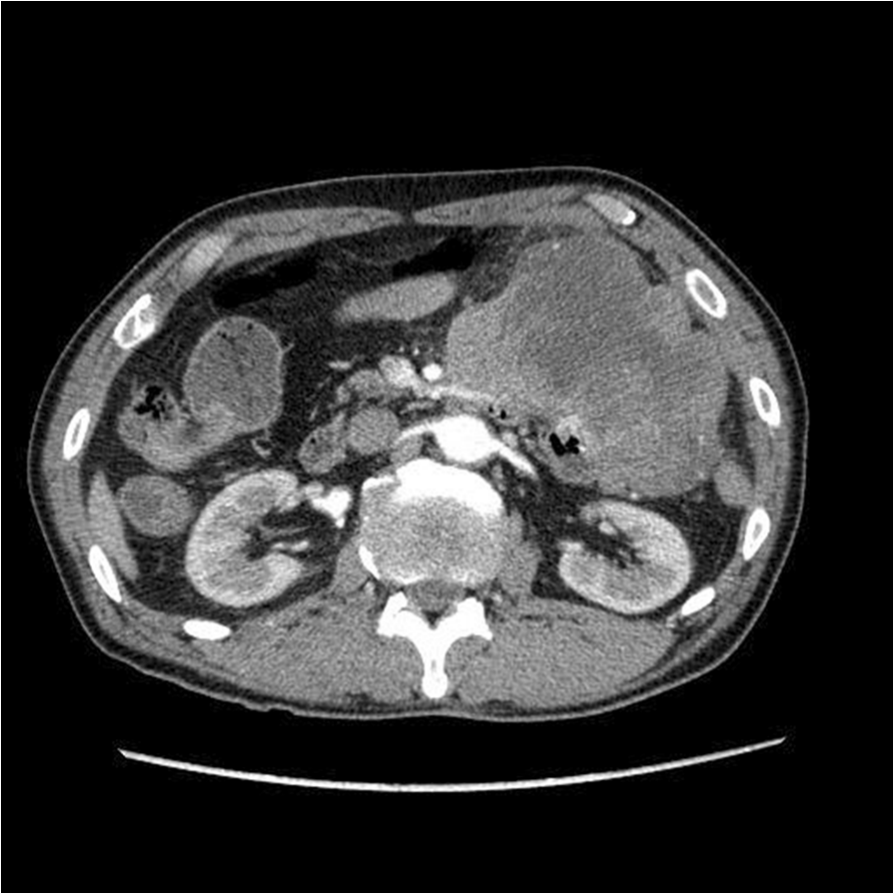
An abdominal enhanced computed tomography scan, revealing a 10 × 10-cm solid tumor with low-density area within, suggesting necrosis or abscess.

**Figure 2:**
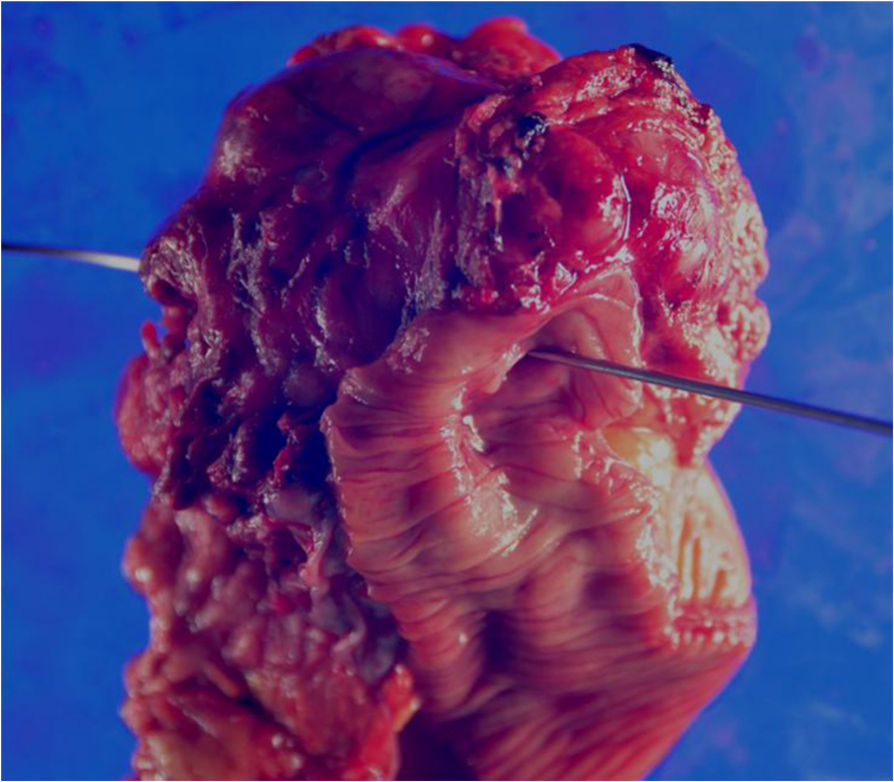
The solid parenchyma contained central necrosis with a fistula to the lumen of the jejunum.

**Figure 3:**
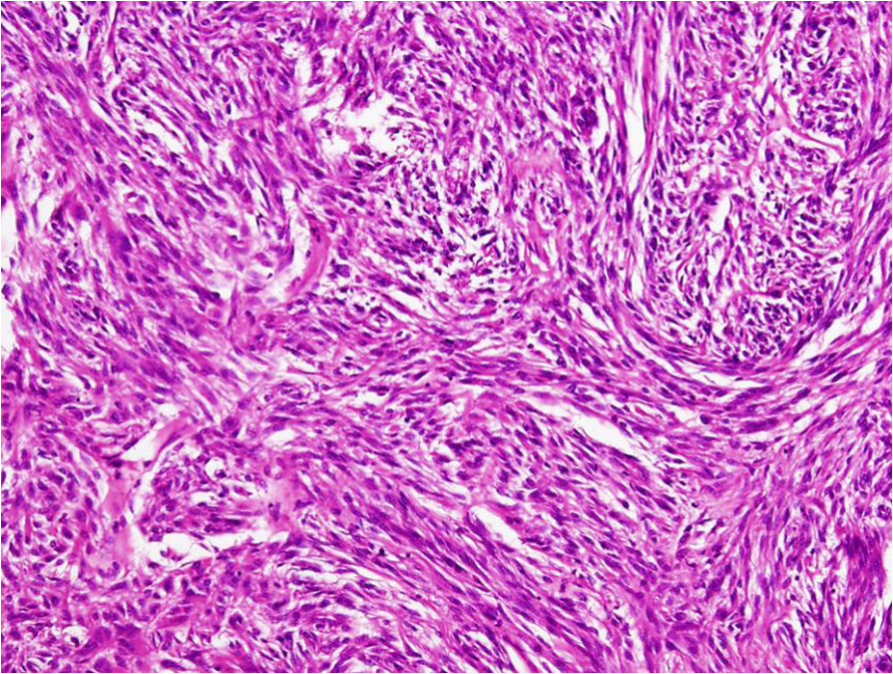
H&E staining showed spindle-shaped cell proliferation.

**Figure 4:**
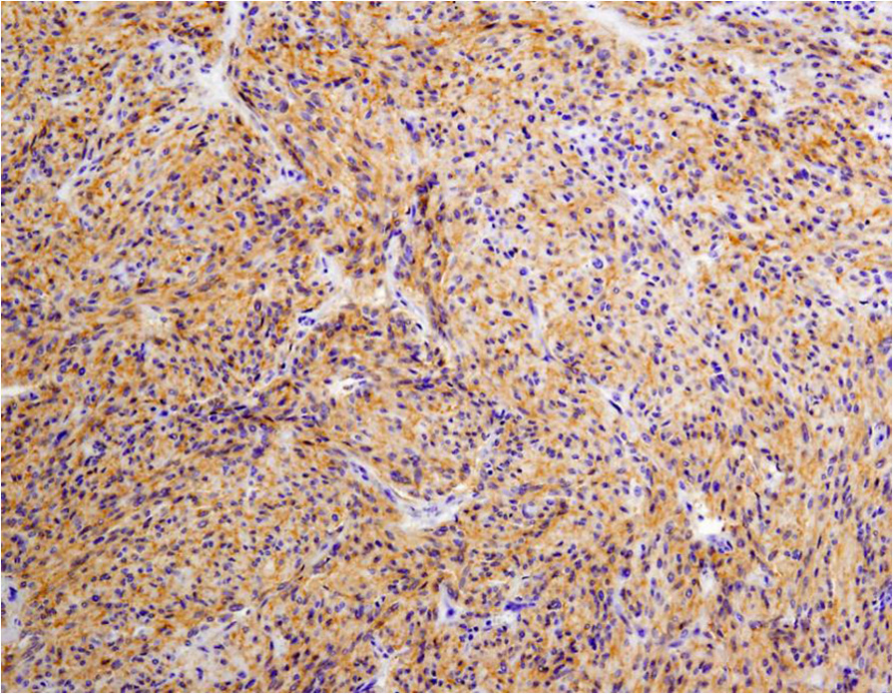
Immunohistochemical staining showed positive results for c-kit.

**Figure 5:**
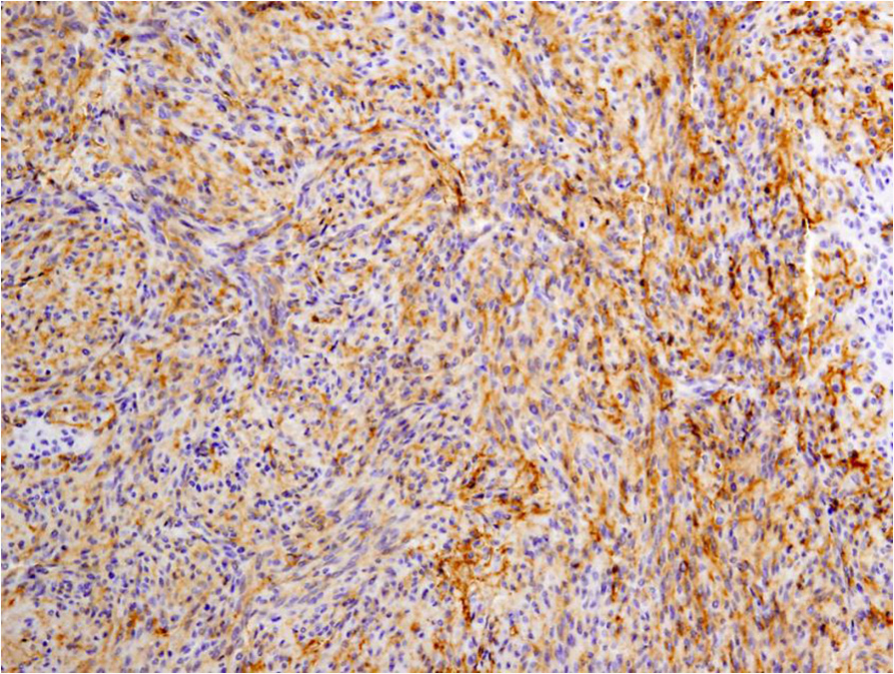
Immunohistochemical staining showed positive results for CD34.

**Figure 6:**
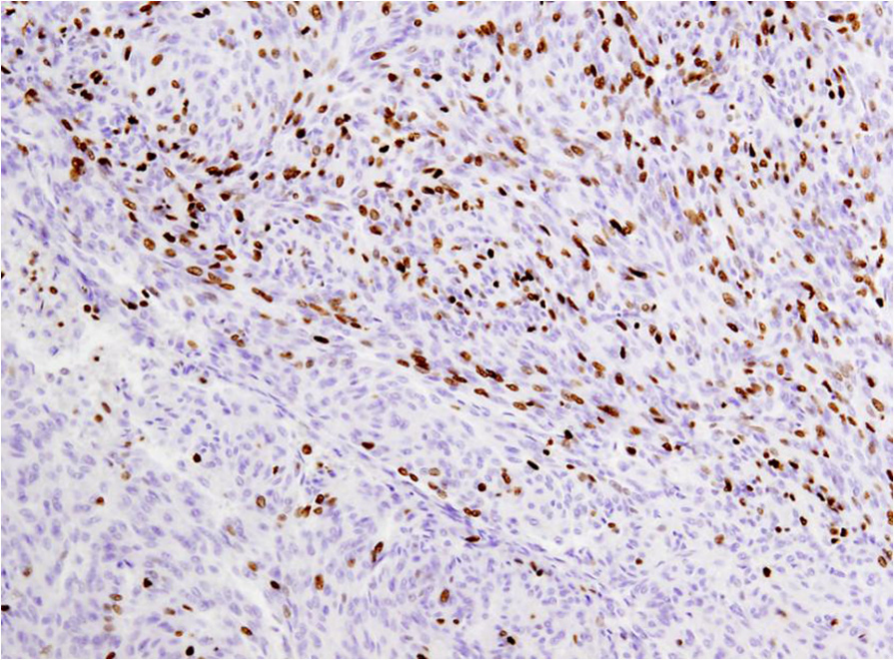
Nuclear expression of the proliferation-associated Ki-67-antigen in approximately 26% of the tumor cells.

A 70-year-old man presented with symptoms of fever and abdominal pain. There were no episodes of gastrointestinal bleeding. The blood pressure was 117/86 mmHg, the pulse 80 beats per minute, and the temperature 38.2°C. Abdominal examination revealed tenderness and muscular defense in the left upper quadrant, and rectal examination revealed an empty ampulla. Blood tests showed a hemoglobin level of 7.2 g/dL, white blood cell count of 25,490/mm^3^, and C-reactive protein of 19 mg/dL. An abdominal enhanced computed tomography (CT) scan revealed a 10 × 10-cm solid tumor with a low-density area within, suggesting necrosis or abscess; additionally, a liver metastasis was suspected (Figure 1). Upper GI endoscopy revealed no aberrations up to the third portion of the duodenum. Examination of small bowel x-ray series revealed no tumor penetration. The mass was suspected as a GIST of the jejunum accompanied by a giant abscess. The patient was treated with intravenous administration of antibiotics and blood transfusion. Subsequent to improvement in his clinical condition and laboratory tests, laparotomy was performed. Intraoperative findings showed mild ascites but there was no blood in the abdominal cavity. We also found an outgrowing 10-cm mass in the jejunum near Treitz’s ligament. The tumor was ruptured and peritoneal metastasis was detected around the tumor. Segmental resection of the jejunum with the tumor was performed. The resected mass was a well-circumscribed tumor measuring 10 × 10 cm and penetrating the jejunum. The solid parenchyma contained central necrosis with a fistula to the lumen of the jejunum (Figure 2). H&E staining showed spindle-shaped cell proliferation (Figure 3), and immunohistochemical staining showed positive results for c-kit (Figure 4) and CD34 (Figure 5), and a nuclear expression of the proliferation-associated Ki-67-antigen in approximately 26% of the tumor cells (Figure 6). GIST (high-risk group) of the jejunum was diagnosed and treated with imatinib mesylate, 400 mg daily, after the 21st post-operative day. The patient was discharged after endoscopic dilation for an anastomotic stricture. Medical treatment was continued and the patient was followed up with an abdominal CT scan 12 months after the surgical procedure without any signs of recurrence.

## Discussion

GISTs are visceral tumors arising from any site of the gastrointestinal tract. Approximately 60 to 70% of cases occur in the stomach, 25 to 35% in the small intestine, and 10% in the jejunum, whereas the esophagus, colon, rectum, and appendix, are rarely affected [4]. Bleeding (30 to 40% of cases) comprises the most common symptom after vague abdominal discomfort. Patients with GIST of the jejunum usually suffer from abdominal pain or have a palpable mass, and also complain of early satiety and abdominal fullness. Pressure necrosis and ulceration of the overlying mucosa may cause gastrointestinal bleeding, and patients who experience a significant blood loss may suffer from malaise and fatigue. Bleeding into the lumen of the GI tract, causing hematemesis, melena, or anemia, is usually more chronic on presentation [5]. Obstruction may result from intraluminal growth of the tumor or luminal compression from an exophytic lesion. Fever, anorexia, and weight loss are rarely observed, and GISTs originating in the jejunum seldom cause perforation and acute diffuse peritonitis. A few cases have been reported in the English medical literature associated with perforation or rupture of GIST in the small intestine [6-16] (Table 1). Bleeding into the peritoneal cavity due to a ruptured GIST can lead to acute abdominal pain presenting as a surgical emergency [6,7,10,12,16]. In addition, only a few cases of GIST of the small intestine accompanied with abscess formation have been reported [9,14]. In this case, GIST originating in the jejunum was invaded via a fistula, leading to central necrosis and spontaneous rupture into the peritoneal cavity. For GISTs, prognostic markers that include tumor size > 5 cm, mitotic rate > 5/50 high-power fields (HPF), tumor necrosis, and a Ki-67(MIB-1) index of > 10% are all associated with malignancy of the tumor and high mortality [17-19]. GISTs can be categorized as low or high risk tumors by taking into account the possibility of metastasis or recurrence. However, the main prognostic factor is the mitotic count. A prognostic classification was defined by Fletcher *et al*. and is widely accepted and used today [17-19] (Table 2). In contrast, the MIB-1 index (>22% in the most active area) was the most powerful predictor of poor survival [20]. The prognosis is dismal when the tumor presents with symptoms or signs such as perforation or rupture, multifocal location, or metastatic lesions. Patients with localized or locally advanced tumors have 46% 5-year survival, except for patients with a metastatic or multifocal tumor. Those patients have 5-year survival at 24%, probably because of peritoneal dissemination [21].

**Table 1:** Summary of spontaneous ruptured gastrointestinal stromal tumor (GIST) of small intestine in the english medical literature

	**Author (reference)**	**Year**	**Age**	**Sex**	**Location**	**Symptoms**	**Diagnosis modality**	**Intra-abdominal bleeding**	**Abscess formation**	**Intra-abdominal dissemination**	**Mitotic count**	**Size (cm)**	**Treatment**	**Outcome**
1	Ajduck M [6]	2004	60	F	jejunum	abdominal pain	laparotomy	+	-	-	3/50HPF	7.5	SR	N/A
2	Cegarra-NavarroMF [7]	2005	76	M	jejunum	abdominal pain	CT	+	-	-	< 5/50HPF	6	SR	31 months ANED
3	Efremidou EI [8]	2006	66	M	ileum	abdominal pain	laparotomy	-	-	-	2/50HPF	7 × 5 × 4	SR + Imatinib	44 months ANED
4	Karagülle E [9]	2008	70	M	jejunum	abdominal pain	CT	-	+	-	0/50HPF	5	SR	13 months ANED
5	Hirasaki S [10]	2008	87	F	ileum	abdominal pain	CT	+	-	-	N/A	13 × 11	SR	16 months ANED
6	Ku MC [11]	2010	33	F	jejunum	abdominal pain	CT	-	-	+	N/A	6.5 × 5.3 × 3.9	SR	N/A
7	Varras M [12]	2010	28	F	N/A	abdominal pain	US	+	-	-	> 5/50HPF	13	SR	3 years ANED
8	Feng F [13]	2011	45	M	jejunum	abdominal pain	CT	-	-	-	< 5/50HPF	10 × 8	SR	N/A
9	Chen HW [14]	2011	22	M	jejunum	abdominal pain	CT	-	+	-	N/A	5	SR	2 months ANED
10	Memmi N [15]	2012	59	M	jejunum	abdominal pain	CT	-	-	-	7/50HPF	12	SR	N/A
11	Nannini M [16]	2013	45	F	jejunum	abdominal pain	laparotomy	+	-	+	2/50HPF	12	SR + Imatinib	13 months ANED
12	This case	2014	71	M	jejunum	abdominal pain fever	CT	-	-	+	MIB > 26%	9 × 9	SR + Imatinib	12 months ANED

*Abbreviations*: *ANED* alive with no evidence of disease, *N/A* not available, *SR* segmental resection of small intestine.

**Table 2:** **Prognostic classification of gastrointestinal stromal tumors (GISTs) by Fletcher *et al*. **[17]

**Risk of malignancy**	**Size of tumor (cm)**	**Mitotic counts (/50HPF)**
Very low	< 2	< 5
Low	2 to 5	< 5
Intermediate	< 5	6 to 10
	5 to 10	< 5
High	> 5	> 5
	> 10	Any counts
	Any size	> 10

The introduction of imatinib mesylate, a tyrosine kinase inhibitor targeting KIT has provided a much needed chemotherapeutic option for patients with both resectable and unresectable GISTs. Despite the noted success of imatinib, surgical resection is the main treatment modality for primary GIST of any localization [22,23]. Imatinib treatment of GISTs is a dynamic process with the permanent risk of pharmacoresistance and maximal response within the first six months of therapy [24]. Futhermore, the patients with a primary GIST treated for spontaneous rupture or with rupture occurring during resection have a very high risk of tumor recurrence. These patients are candidates for adjuvant treatment with imatinib [25]. Our case is classified as high-risk GIST with poor prognosis and medical treatment was continued with imatinib; however, no signs of recurrence were observed with abdominal CT scan 12 months after the surgery.

## Conclusion

In conclusion, spontaneous rupture is a rare presentation of GIST and preoperative diagnosis is difficult because of the absence of pathognomonic signs or symptoms. The diagnosis should be suspected whenever there is a presentation of fever and abdominal pain in patients with an intraabdominal mass. In this case, GIST originating in the jejunum led to central necrosis and spontaneous rupture in the peritoneal cavity; however, local excision of the tumor associated with adjuvant therapy with imatinib mesylate was effective and these treatments remain the main modality of treatment of high-risk GISTs.

## Consent

Written informed consent was obtained from the patient for publication of this report and any accompanying images.

## Abbreviations

CT: computed tomography; GIST: gastrointestinal stromal tumor; H&E: hematoxylin and eosin.

## Competing interests

The authors declare that they have no compating interests.

## Authors’ contributions

SM was responsible for the writing. MT, HS, YK and HT participated in the clinical management of the patient. HO carried out the pathological examination. All authors read and approved the final manuscript.
